# “What is Wrong With You People That You are Happy Someone has Covid” Impoliteness in the Coronavirus Pandemic Era

**DOI:** 10.1177/21582440231161040

**Published:** 2023-03-15

**Authors:** Jean Mathieu Tsoumou

**Affiliations:** 1 Universidad Europea de Canarias, Madrid, Spain

**Keywords:** Facebook, impoliteness, politics, polarized discourse, Covid-19

## Abstract

Whilst the coronavirus pandemic keeps threatening the world at large, little is yet known about the impoliteness implications of user-generated- Covid-related contents on social media such as Facebook. The aim of this study is to examine the comments made in response to Giuliani’s Covid- 19 diagnosis, from an impoliteness perspective. A merely qualitative analysis of a dataset of 3,000 comments evenly collected from three different news outlets (i.e., BBC, CNN, Fox News), the findings reveal that the reactions to Giuliani’s diagnosis are more focused on him being a politician, than him being a human being affected by the virus. The reactions attack his actions and the actions of others within his political party, which suggests that impoliteness has a strong dependence on previous actions and political engagement. Giuliani is seen by some users to be undeserving of compassion or empathy not just on the grounds of his active involvement in attempting to overturn the presidential election results, but also for his disregard toward mask wearing in public spheres. Not all the users, however, appreciate the attacks against Giuliani. Through metadiscursive comments, some users not just feel the need to treat Giuliani as a human being, but more importantly remind fellow users that Covid-19 should be a concern for all. What is particularly critical about these metacomments is that while the users advocate for civil interactions, they mostly do not condone Giuliani’s actions. This so because these users understand what should be obligatory, permissible, or forbidden on the human level under the circumstances.

## Introduction

The year 2020 is best remembered as the year of the outbreak. Detected in China in late 2019, Covid-19 soon became an international emergency by March 2020. The damage brought about by the outbreak became a hardship felt worldwide, as lockdowns became the norm in most countries globally. The management of such a disastrous pandemic tested the world nations’ leaderships and set the tone for political discourse over issues such as mask wearing, test distribution, length of lockdowns, health recommendations and guidelines, travel restrictions, etc. In the USA where 2020 was also known as the presidential election year, the abovementioned issues became even more salient as they resurged the conflicts between traditional and progressive values which have long divided Democrats and Republicans (Garcés-Conejos Blitvich, 2010). For example, whilst Democrats were more open to and welcoming of mask wearing, Republicans took a reluctant stance toward the issue, making the US response to the outbreak one of the most controversial and polarized issues from the earlier days of 2020. Then-conservative President Trump even downplayed the pandemic as early as February 2020, called it a hoax and barely wore a mask throughout 2020, which led the Democrats to see in the pandemic an opportunity to criticize the Republicans and energize their electoral base. From the start of 2020 to its end, Covid-19 in the US went from a merely public health issue to becoming a political challenge. Being a superpower struggling to contain the virus while the death toll keeps skyrocketing across the country (https://www.cdc.gov/coronavirus/2019-2019-ncov/covid-data/covidview/pastreports/11062020.html), the US response to the outbreak and its implications ahead of the US presidential election scheduled for November 3, 2020, caught the attention of the rest of the world both through international news outlets such as BBC, France 24 as well as on social media such as Twitter, Instagram, Facebook, etc. Millions of people worldwide carefully followed the pandemic crisis as it unfolded on social media and live TVs. Contents and statistics on the devastation induced by the virus became constantly available and reported live on both traditional news stations and social media. In the particular case of social media, for instance, the more alarming the pandemic got, the more polarizing the reactions became, especially when it came to prominent politicians being diagnosed with Covid-19 (Tsoumou, 2022).

Within impoliteness literature, polarization implies, according to Andersson (2022, p. 490), “contradiction (verbal and non-verbal) attacks, criticisms, etc., which sometimes violate the norms of appropriate social behavior (or even civility), create an atmosphere of negative emotion, and attack the participants’ self-image and/or social identity” (See also Culpeper, 2011; Tsoumou, 2022). However, the extent to which such polarization shapes individuals’ reactions to politicians’ Covid diagnosis as well as the grounds on which they base such reactions on social media such as Facebook have not been fully explained from an impoliteness perspective. As such, this paper examines impoliteness in comments reacting to Giuliani’s COVID diagnosis on Fox News, CNN, and BBC Facebook pages. It aims to understand how impoliteness unfolds and the grounds on which it is based. The data were collected on December 6, 2020, while Giuliani (full name “Rudolph William Louis Giuliani”) was the Attorney to the then-President of the United States, Donald Trump. Giuliani was one of the lawyers appointed by Trump to challenge the 2020-US-November-3rd-election results. Besides, Rudy Giuliani has been in the public eye for countless years, including being the New York City mayor between 1994 and 2001, launching a campaign for the US senate 2000 and for the presidency in 2008. Rudy Giuliani is and has been a Republican for many years. As I argued elsewhere (Tsoumou, 2022, p. 12), “at the time of a COVID-19-induced pandemic as well as a looming presidential election, any positive COVID-19 test results of the Trump’s allies had both political and public health implications and ramifications.” This makes Facebook interactions about Rudy Giuliani’s diagnosis a suitable set of data for the analysis of impoliteness. Consequently, this paper intends to provide empirical responses to the following questions.

- How does impoliteness unfold in Facebook comments triggered by Giuliani’s diagnosis?
- On what grounds are the reactions to the diagnosis based across the three news outlets?

The remaining sections of this paper are organized in the following way. The overview of the literature is provided in section 2. The research method is explained in section 3. The analysis of the findings is carried out in section 4. The conclusions drawn from the analysis are provided in the last section.

## Overview of the Literature

The consensus in the literature is that social media such as Facebook have the capacity and power to shape political and health discourse and attract new forms of engagement (Tsoumou, 2020, 2022; Velasquez & Rojas, 2017). The popularity and benefits of social media, however, come with interactive costs and challenges related to the norms of interaction, given that interactions about topics such as politics tend to promote interactional friction and impoliteness (Andersson, 2021; Tsoumou, 2022). In other words, participation on social media, especially when the interaction occurs in international contexts, poses the challenge over what is (in)appropriate. One form of conduct may be judged appropriate by some, but offensive by others. Another point that is often raised in the literature is that social media tend to have a strong emotional focus which makes them prone to all forms of expressions, including compliments, grief, appraisal, mourning, condolences, etc. (Oz et al., 2017; Rossetto et al., 2015; Theodoropoulou, 2015; Wagner, 2018; Zhou & Jurgens, 2020). Since impoliteness can be linked to emotion (Culpeper, 2011), interaction on social media can help understand better, as Tsoumou (2022, p. 2) puts it, “the virtual nature of individuals’ apprehension and management of emotionally charged topics such as death, illnesses, natural catastrophes, as well as other life-changing experiences such as weddings, birthdays, graduations, etc.” However, despite this growing interest in analyzing users’ reactions on social media, very few studies have attempted to examine reactive responses to an emotionally charged topic (i.e., illness) in a polarizing context (Tsoumou, 2022). This paper hopes to contribute toward redressing this imbalance by analyzing reactions to Giuliani’s Covid diagnosis across three international news outlet Facebook pages, namely Fox News, CNN, and BBC Facebook pages.

### Facebook Interaction and Political Discussion

Thanks to its affordances, Facebook has become a popular social media to express not just distress and achievements which usually prompt support, wishes, appraisal, etc. (Li et al., 2019; Zhou & Jurgens, 2020), but also political engagement, criticisms, threats, etc. As Tsoumou (2022, p. 4) points out “while emotional support is commonplace on social media, so are conflictive and impolite interactions, especially in polarizing and politically-driven exchanges where the dissociation from others is a substantial part of a polarized positioning.” As pointed out earlier, the question over what is socially (in)appropriate on Facebook is often a dilemma for users involved in discussions about controversial topics (i.e., politics). The argument usually made is that the notion of politics involves groups in adversarial relationships, and to advantage one group often by definition will disadvantage the other group (Andersson, 2022; Tsoumou, 2022). However, it is not my argument here that every political discussion on Facebook revolves around impoliteness (attacks, criticisms, etc.), as Facebook can also serve as a social media on which opinions about politics may contribute to enhance political discourse. Moreover, attacks in politically related discussions are not just present on Facebook alone. Research shows that other social media such as YouTube, Twitter, etc., are also home to impoliteness. Lorenzo-Dus et al. (2011), for instance, not only report dissociating cases where the users dissociate from others, they also uncovered an emergence of “(sic) larger and more transient subgroups emerged therein, whose members did not always make explicit the distance existing between them and (an)other member(s)/sub-groups” (p. 2587).

Furthermore, researchers tend to concur over the fact that while posts on Facebook intend to call for reactions (be it verbal or non-verbal) from fellow users, such reactions are often influenced by various factors such as the context of the exchange, the topic of conversation or even the type of audience (Tsoumou, 2020, 2021a). For example, it has been shown that polite and friendly reactions tend to be pervasive in interactions and posts about birthday wishes, and condolences, whereas reactions to topics such as gun control, politics, and sports tend be more hostile and impolite (Oz et al., 2017; Theodoropoulou, 2015; Zhou & Jurgens, 2020). Concretely, Zhou and Jurgens (2020) report that tweets reacting to White House posts were more impolite regarding same-sex marriage than there were about other issues such as gun control.

Previous studies reiterate the argument that impoliteness revolves around the notion of norms which can be cultural or situational (Culpeper, 2011; O’Driscoll, 2020). According to O’Driscoll (2020), cultural norms refer to the totality of individual’s experiences of a particular culture and situational norms refer to the totality of individual’s experiences of a particular situation in a particular culture. However, the situational norms are not just part of the cultural norms, but together, they amount to a person’s knowledge of what is normal in certain kinds of situations in a certain culture (O’Driscoll, 2020). Studies on social media posts and subsequent reactions at individual nation level emphasize the situatedness and cultural nature of criticisms, verbal attacks within specific national borders, even when the topic of discussion has international ramifications. Andersson (2022, p. 509), for example, points out “the pertinence of value positions and the participants’ efforts to enact, negotiate, and demonstrate their allegiance to specific values when reacting to the pandemic of Covid-19 on the official Facebook page of the Swedish national public television.” In the context of Spanish politics, Bou-Franch (2021, p. 290) reports that “the meanings of disrespectfulness and childishness in political debates are reported to be connected to breaches in appropriate behavior regarding turn-taking and topic management and social expectations associated with the role of political leaders.”

However, one cannot overlook the fact that when facing global challenges such as the Covid-19 pandemic, global warming, etc., the tendency is often for some nations to take example of how-to response from superpower countries. This usually generates international interest to countries that want to appeal their responsive actions to the superpower models. For instance, the interest the American experiment has had around the world is undeniable. The idea that America rests on solid and permanent democratic principles that guarantee freedom and protection of basic rights for all is usually seen as the right pathway for allies around the globe, especially in Europe. It is in this context that the US struggles to contain the virus while the death toll kept skyrocketing across the country in 2020 and the implications of the US response to the outbreak ahead of the US presidential election scheduled for November 3, 2020, not just generated interest to the rest of the world, but favored international engagement in *translocality* digital environments. Defined as “the complex ways in which diverse local practices come together in global spaces” (Garcés-Conejos Blitvich & Bou-Franch, 2018, p. 5), *Translocality* environments are, for example, Facebook pages held by international news outlets such as the BBC, CNN, FOX News, etc. These pages are open internationally and capable of reaching communities with different languages, backgrounds, cultures, histories and whose members only come together online with the sole interest of informing and being informed of what is going on around the world. Investigating norms and the particularities of interaction in these *translocality* Facebook environments could further our understanding of impoliteness beyond national borders.

### Impoliteness as a Social Practice

Impoliteness research has witnessed a discursive turn in the last two decades. This has evolved toward analyzing longer fragments of authentic discourse with the premise that impoliteness is a social practice carried out through (conversational) assessments made by interactants on the grounds of shared moral order (Haugh, 2013). Haugh and Kádár (2013, p. 6) argue that the basis of impoliteness evaluations is “the accountability of social actions and pragmatic meanings vis-à-vis the moral order,” which is understood as a “set of expectancies through which social actions and meanings are recognizable as such, and consequently are inevitably open to moral evaluation.” In order words, as individuals, we recognize and know the difference between good and bad. Our reaction to any transgression of social norms rests on the knowledge of the form and content of the transgression. Such reactions represent our perceptions and apprehension of social actions vis a vis the moral order. We take other people responsible for their actions on the grounds of what is morally (in)acceptable or (in)appropriate in a particular context. Viewing (im)politeness as a social practice implies that (a) (metapragmatic) evaluations constitute reactions that strongly depend on prior social actions of the person or conduct being evaluated; (b) any (metapragmatic) assessment is rooted in the moral order that orient individuals’ action to deontic order (i.e., what participants think is “obligatory, permissible, or forbidden)” (Stevanovic & Peräkylä, 2012, p. 289).

The term evaluation is crucial when defining impoliteness as a social practice, since impoliteness behaviors depend on the perception, experience and evaluation by not just individuals involved in the interaction alone, but anyone close enough to experience such behavior. Evaluation is what gives an utterance a positive or negative value. Put differently, what makes people perceive particular behaviors as impolite is the value accorded to them in a particular community (O’Driscoll, 2020) and the salience of this value in a given interaction in a particular context.

However, when examining impoliteness, one has to be aware of not just the importance of the hearer’s evaluation (Eelen, 2001), but also the alignment individuals take up to themselves and others present as expressed in the way they manage the production and reception of an utterance (Haugh, 2013). For example, the perception of Giuliani as deserving of compassion or not is both socially and interactively meaningful, since the production of any ((im)polite) comment in this context rests on the premise that this utterance will actually be perceived as such not just by the addressee alone, but all those involved (maybe involved) in the interaction.

Yet, individuals’ evaluation of social actions, especially on Facebook—which is a polylogual interaction—depends on the participation status of the participants, also known as footing (Goffman, 1981). Footing refers to the roles and responsibilities of participants or the social capacity in which a participant is presumed to be acting in the interaction (O’Driscoll, 2020). According to Haugh and Kádár (2013), these roles and responsibilities include ***animator*** (or utterer) which refers to the person producing talk, an ***author*** is the entity that creates or designs the talk, a ***principal*** which is the party responsible for that talk, and a ***figure*** is the character portrayed within the talk. These roles and responsibilities may affect the perspectives from which impoliteness is evaluated and perceived. For example, an animator may produce an utterance that is only perceived as impolite or offensive by the author, rather than by the intended target. Put differently, social media (i.e., Twitter and Facebook) have a policy based on which they sanction offensive behaviors not because the targets of such offenses complain, but because these acts go against the policy of the platform. Trump, for example, has been banned from Twitter for violating the platform policy.

The notion of footing is particularly salient in the case of the data analyzed in this paper, as these roles can broadly be distributed as follows: the animators are the users engaged in the interaction the comment threads, the author here is Facebook, the principals are the three News outlets and the figure is Giuliani who is portrayed within the talk. In this context, while author, principal, and figure seem to have a static and unique role, the animators can be categorized into those that produce impoliteness utterances (producers of comments) and those that morally see and evaluate these utterances as impolite (producer of metacomments). Evaluations of impoliteness in this context needs to be situated vis-à-vis not only simply speakers or hearers, but also relative to a complex array of production and reception footings (Haugh, 2013).

Time and space are also of particular importance in understanding impoliteness as a social practice in the sense that evaluative moments within a particular interaction are usually underpinned by the different understanding of time and space (Haugh & Kádár, 2013). In other words, impoliteness evaluations are usually situated. Such situatedness is conditioned by time and space. Any understanding of impoliteness is therefore relative to time and space. In this way, impoliteness evaluations are practices that emerge in an interaction in a particular period of time. This is critical to the present paper in that the interaction under study takes place in a particular space (Facebook) and during a particular time (the Covid-19 pandemic and the US post-election period). In other words, analyzing impoliteness in Facebook interaction dealing with Covid-19 is, in other words, a search to determine the salience of impoliteness as it emerges and its motive values in the context of the 2020 pandemic.

### Detecting Impoliteness Across the Corpora

Recent developments in impoliteness research have shown that impoliteness behaviors can be interpreted from the scientific standpoint (second order), from lay persons’ standpoint (first order) or from a hybrid approach, which is the combination of the above (Bou-Franch, 2021; Garcés-Conejos Blitvich, 2022). This distinction sparks from the mere idea that impoliteness behaviors are contested social actions which can be differently understood and interpreted (Haugh & Kádár, 2013; Kleinke & Bös, 2015). Taking each Facebook comment as a unit of analysis, I examine impoliteness by combining a metapragmatic analysis with a merely linguistic analysis so as to guarantee a hybrid approach which benefits from Culpeper’s (2011, 2016) notion of impoliteness formulae (second order) and lay users’ interpretation of impoliteness (first order). In other words, a comment is deemed impolite not just by the way it is discursively constructed and linguistically formulated, but also by the (re)evaluation it receives in the flow of the interaction across the three corpora. The task is to read each comment in each corpus and systematically identify these formulae that operate as “multiword collocations which are stored and retrieved holistically rather than being generated de novo with each use” (Kecskes, 2016, p. 62). For example, you are stupid is a formulaic expression of insult (Culpeper, 2011). Impoliteness comments are not just acts that threaten or attack the addressee, and the idea behind conventionalized formulae is, as O’Driscoll (2020, p. 18) argues, “that by virtue of people’s experience of the regularity of their occurrence in particular co-texts and/or metadiscourse around (im)polite language, they come to be associated with impoliteness in people’s minds.” These formulae, which are usually semantically tagged for context (Culpeper, 2011), are prepackaged expressions which are readily available as a means of causing offense and, by the same token, are comparatively readily interpreted as offensive. Insults, criticisms, threats, silencers, dismissals are examples of conventionalized impoliteness formulae (see subsection 4.1.).

Finally, whilst Hampel (2015, p. 104) argues that “individuals’ perceptions of appropriate or polite behavior also play a vital role since differing expectations and interpretations of (im)politeness may lead to conflicts”, Haugh (2013, p. 58) emphasizes that “the evaluation is one way to study impoliteness”, since, “impoliteness occurs not so much when the speaker produces behavior but rather when the hearer evaluates that behavior.” With this in mind, a thorough metapragmatic examination of each corpus is carried out so as to determine users’ own evaluation of the ongoing flow of interventions within the comment threads (see subsection 4.1).

## Method

**Figure 1.**
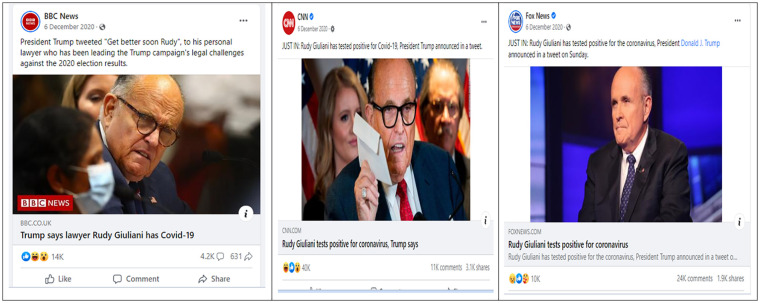


It is now widely accepted that any investigation into human behavior on Facebook requires a reflection on the questions of privacy, confidentiality, and informed consent (Bolander & Locher, 2019). As a result, precautions have to be taken in order to avoid inflicting harm to individuals being investigated. In this vein, the research design in this paper carefully follows the guidelines for online research (Spilioti & Tagg, 2017) in accordance with calls for attention to ethical considerations in pragmatics and social media research (Bolander & Locher, 2019). In this regard, 6 months prior to the collection of the data analyzed in this paper, I had been conducting a field observation of the Trump administration handling of the pandemic and its implications in the 2020-US elections. To narrow down this task, I carefully selected three Facebook pages belonging to two competitive news outlets within the USA (Fox News and CNN) and one foreign news outlet (BBC News). Part of the data—that is, the reactions from BBC Facebook page—has already been published in separate paper (See Tsoumou, 2022). When I first started the observation process, I reached out to the administrators of these pages to express my interest in studying the comments on their pages. Therefore, I did not filter the comment threads or single out irrelevant comments for the simple reason the purpose of the study is to analyze naturally occurring data. In this context, the news about Giuliani testing positive was initially announced via Twitter by Donald Trump, whom I personally followed on Twitter. Therefore, as soon as I got the notification of Trump’s tweet about Giuliani’s positive test, I logged onto the abovementioned Facebook pages, and as soon as Fox News, CNN, and the BBC reported the news on Facebook, I decided to start the collection process while observing how the interaction unfolds and how the interplay between politeness and impoliteness becomes salient and worth investigating. For the sake of fairness, I evenly collected 1,000 first Facebook comments from each of the three abovementioned pages (see Figure 1) on December 06, 2020. As the posts went viral in just 4 minutes with over 14K likes and thousands of comments, all the corpora were collected within the first 30 minutes of the publication.

It is important to point out the motive for choosing these three news outlets. Based on the traditional political standings, Fox News page is a pro-republican, pro-conservative, and pro-Trump news page. CNN is a pro-democratic, pro-liberal, and almost anti-Trump news outlet. Finally, as a foreign TV broadcast, the BBC is neutral when dealing with US-related issues and would generate an equilibrium of between well-wishing and ill-wishing comments. In this context and since impoliteness implies verbal attacks and criticisms, one may attempt to argue that there is likelihood for users to act more antagonistically on the CNN Facebook page than on the Fox News one. However, this expectation may be misleading on the grounds that we may assume that we know where the updates are posted from, but it is hard to determine where the reactions are posted from. These three Facebook pages have a strong outreach which goes beyond the national borders and situate them at an international scale which, one may argue, defies nationally known political standings. Moreover, unlike Facebook pages held by politicians which often encourage the participation and engagement of followers or users with clear political and party-line commitment (Tsoumou, 2020), pages held by news outlets are consulted by a broader audience, which sometimes has no specific political allegiances. Therefore, in the absence of self-reported information—which I could have gathered through interviews with the users—I avoid exploring the data with the presumption that Facebook users reacting on the Fox news page are all conservative and those on CNN pages would have progressive values. For this reason and for the sake of space, this paper will only carry out a qualitative analysis while leaving a quantitative analysis of findings as well as the examination of the users’ political standings for future research. After all, impoliteness is about the quality of the interaction, rather than the frequencies of actions. On the other hand, keeping in mind that Facebook algorithms have the power to arrange comments, the 3,000 Facebook comments were gathered in a chronological manner from the oldest to the most recent. The process of data collection consisted of copying and pasting the comments onto a .doc file.

Understandably, there are a number of drawbacks as well as advantages about this type of data. One issue about the nature of these data is that (1) the protagonist—that is, Giuliani—is absent from the development of the conversation within the Facebook thread, (2) the researcher bears no physical contact with the participants. Additionally, the copying and pasting of the data, identification and classification of the comments were carried out in a pen-and-paper fashion. One of the methodological advantages about this type of data, however, is that the data are readily available and can, as a result, be easily and conveniently collected. This easiness consisted of simply copying the comments from the news outlet page and posting them into a .doc file. Another advantage is its accessibility. To the day of the writing of this paper, the data source remains available to the public. Moreover, what is particularly important about this type of data is that it provides the researcher with an opportunity to carry out a real time investigation of three different sets of interactions dealing with the exact same issue at the same time, which otherwise would be impossible.

As for ethical considerations, Bolander and Locher (2019, p. 85) insist that “in an attempt to not cause our interlocutors harm – an intent which is at the heart of ethically sound research – we must be mindful of the complexity of the research process, and do our level best to reflect upon the best ways to work with data without causing harm to our interlocutors.” In this vein, the data for the present paper were gathered without the researcher’s participation in the on-going interactions and without previously informing the participants. My participation was merely observational. In addition, it is true that online data remain a source of long-time debate as regards their nature, as some scholars consider online materials to be fundamentally private, and copyright protected which requires researchers to give credit to the copyright holders by eventually asking them for consent as far as the copyright law is concerned (Villi & Matikainen, 2016). There are, however, scholars who counter argue that any materials left online are public. As such, they can be used without asking for any consent from owners (Kozinets, 2015). In this paper, I follow this second line of ideas. Therefore, given that the comments here analyzed were posted for public display, no consent was sought from the users prior to the data collection. In fact, as mentioned earlier, to the date of the writing of this paper, the data remain publicly available on the Facebook pages of the news outlets. Nevertheless, I ensured that all the details related to the users’ identities be removed in the analysis. Likewise, I employed **U** to represent both the user and the rank of their interventions within the threads in each corpus. Throughout the analysis, the findings are presented in the following order. Comments from the BBC (U_BBC_) appear first in each excerpt analyzed, followed by comments from CNN (U_CNN_). Comments from FOX News (U_FOX_) are presented last.

Finally, a multimodal analysis is critical in carrying any investigation on social media, especially Facebook. This rests on the argument that multimodal and mainly textual discourse are inseparable and emotionally charged in digital discourse. Studying impoliteness implies analyzing individuals’ emotional representation of social actions. With this in mind, I meticulously investigated all instances where users hashtag fellow users within the interaction so as to determine items directed at Giuliani’s diagnosis and those used as reactions to the comments previously made by fellow users within the threads. When a user addresses a fellow user by means of a hashtag, this is displayed within parentheses in the analysis. Likewise, all the excerpts are reproduced here as they were naturally uttered by the users with errors and infelicities so as to keep their naturally occurring forms. Finally, I took into account the display of emojis in the analysis.

## Results and Analysis

The analysis of the results will first start with a second-order examination of impoliteness as it manifests across the corpora, before elaborating on the first-order perception that arises therein.

### Determining Impoliteness Strategies Across the Corpora (Second Order)

There are multiple ways to act impolitely in a particular interaction. In this subsection I take a second order approach so as to examine the linguistic items used in expressing impoliteness. In excerpt 1, for instance, U_BBC_198 refers to Giuliani as an ***idiot*** Lawyer; U_CNN_201 used ***morons, idiots***, and ***con*** to describe the Republican leadership; U_FOX_624 goes even further as to call fellow users ***evil, sickos, and nasty***.

Excerpt 1:

U_BBC_198: Poor Public that was exposed to the ***Idiots*** Lawyer.

U_CNN_201: Dear Republicans, It must be really embarrassing to call yourself a republican at this point. ***Morons, idiots, and con men, make up the Republican “leadership***.” Time to jump ship and salvage what’s left of your dignity.

U_FOX_624: There are some **very EVIL** people in this world. . I can’t stand Obama, Harris and Biden but I don’t wish them illness either…**SICKOS! Nasty, nasty** □ people.

The disregard in excerpt 1 intends to scornfully abuse, downgrade and control the addressee. It becomes offensive and defiance of expectations given both the fact that the interaction is about a matter of life or death—which morally requires compassion—as well as the fact that the comments obey conventionalized impoliteness formulae associated with offensiveness by virtue of their preponderant use for causing offense (Culpeper, 2011). The choice of linguistic items (i.e., idiot, moron, con, evil, sickos, and nasty), otherwise known as offense generators (O’Driscoll, 2020), shows a deliberate desire to cause pain, humiliate the target and implicitly establish a sort of dominance at the expense of others. What is commonly shared about these comments is, however, that they start by a poor evaluation of previous actions from which the insulting remarks become the consequences of. In other words, they are not initial actions by themselves. For instance, U_BBC_198 starts by expressing his sorrow about anyone who may have been in closer contact with Giuliani whom U_BBC_198 called an idiot. U_CNN_201 takes aim at the conduct of the Republicans vis-à-vis the pandemic. U_FOX_624 is critical of fellow users engaged in the interaction whom the user refers to as ***sickos*** and ***nasty***. U_FOX_624 sees the impropriety of the attacks targeting Giuliani. U624 disapproves negative comments that target a fellow being affected by the virus regardless of the divergence of point of views. Being the results of previous actions, these acts all involve pointing out some kind of fault (i.e., weakness, failing, misdemeanor, or mistake), and expressing disapproval of that fault (Haugh & Chang, 2019). Whether they are genuine or not, the negative evaluations and fault finding in excerpt 1 rest on the moral order that stems from the idea that “being in closer contact with people while maskless” is an irresponsible act during the Covid-19 pandemic or “laughing at someone in pain” is antisocial.

However, since impoliteness is a contested social phenomenon, whether the insults, described here based on a second-order standpoint, are perceived as such requires first-order interpretations or reactions from the targets or anyone capable of seeing such offense. This will be dealt with in subsection 4.2. From now, the focus will be on exploring other second-order manifestations of impoliteness found across the corpora.

In excerpt 2, for example, U_BBC_251 utters a poor evaluation of the Trump presidency, describing it as a 4-year-failure, before comparing Trump to a chimpanzee. Likewise, U_CNN_118 takes aim at the administration by negatively evaluating the quality of the job done. Finally, the criticism uttered by U_FOX_530 comes in a form of complaint, as the user believes that the rates of Covid-19 infections are politically one-sided, as it seems strange to this user that only Republicans have come down with the virus so far. This is a way to create room for speculation on a conspiracy against Republicans and potentially create doubt about the existence of the virus.

Excerpt 2

U_BBC_251: there’s just been 4 years of a **disaster**. A chimpanzee could do a **better job than** Trump

U_CNN_118: Pretty much the whole administration got it, **speaks volumes** about their indifference and where we are at.

**U_FOX_530: Very strange it’s only Republicans that get sick**

The orientations of the pointed criticisms across the three corpora are revealing in the sense that the users in the BBC and CNN tend to attack Trump and his administration, whereas most acts of criticisms in Fox news are uttered in a form of complaints and accusations directed at the democratic party. However, what is commonly shared is that all criticisms are a way to single out an addressee’s fault or wrongdoing while soliciting reasons for the state of affairs for which the addressee is held responsible. All of this is done not just to cause hurt feelings, but also convey expectations that the target will do something to remedy the fault, pass moral judgment on others, or display claims to have expertise or knowledge about the target that may be resisted by the latter in (Haugh & Chang, 2019).

The orientations of the comments here emphasize the argument that being at once a politician and an attorney to the then-President of the United States, Giuliani’s diagnosis cannot be detached from the management of the pandemic (or the lack thereof) from the sitting administration. In excerpt 3, for instance, U_BBC_449 and U_CNN_151 rhetorically implies that Trump is untrustworthy and no one should rely upon him for accurate news, whereas U_FOX_85 raises suspicions around the fact that only Republicans have come down with the virus so far (as was the case of U_FOX_530 in excerpt 3). The interests of these items are not Giuliani’s health; rather, these acts serve the political interests of the participants in the interaction.

Excerpt 3

U_BBC_449: “Trump says???” Couldn’t you find a reliable source?

U_CNN_151: Why is trump announcing that? Why would anyone believe him! There are many things wrong with Rudy that’s evident!

U_FOX_85: How come none of the prominent DEMONCRAPS In DC or anywhere else don’t get Covid?????? Has anyone asked that question?? Kind of fishy if you ask me □

The questions in this except index the clash of priorities of the users across the three corpora, as BBC and CNN users mainly attack the credibility of Trump and his administration with respect to the information they release to the public, whereas from Fox news users tend to drag Democrats into the reason the virus only affects Republicans, even if the disproportion in terms of infection rates is relatively understandable given that the Republicans were reluctant to implement some and appeal to Covid-19 measures such as mask wearing.

In excerpt 4, U_BBC_238 condescends to Giuliani as unstable and incoherent. Likewise, U_CNN_555 treats Trump administration officials as hard-learner individuals. Finally, U_FOX_189 describes Giuliani as a disgraceful person.

Excerpt 4

U_BBC_238: Probably explains why Giuliani has been making less sense than normal lately.

U_CNN_555: They don’t seem to be learning anything!!! / U_CNN_574: That’s what happens when you are reckless!

U_FOX_189: One of America’s most toxic men. Rudys fall from grace is beyond pathetic

Describing Giuliani as *making less sense*, *a difficult learner* or a *toxic person falling from grace* is undoubtedly a takedown. Regardless of the perspective these comments are approached, referring to someone battling Covid-19 as “*making less sense than normal lately*” or calling someone “*reckless*” or “*toxic*” is far from being a positive description of Giuliani. The same argument can be made for excerpt 5 in which U_BBC_726 indirectly orders Republican politicians to wear masks, U_CNN_877 makes an order for Giuliani to be isolated, and U_FOX_35 asks Giuliani to stay home.

Excerpt 5

U_BBC_726: Let your GOP folks to mask up 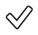

**U_CNN_877: Get him isolated and trace his close contacts**

**U_FOX_35: stay home since you felt it was not important enough to mask up**

The polarization of views over the issue of mask wearing becomes the grounds on which these comments are uttered, as the users enforce the idea that Giuliani’s own irresponsibility for not wearing masks is what opened the door for him to catch the virus, and if he does not isolate himself, he may end up spreading the virus further. However, although masking up can be beneficial to Giuliani in terms of preventing or fighting the virus, ordering GOP folks (which includes Giuliani) to mask up is certainly a form of condescending behavioral practices through which the users attempt to patronize the interaction. The use of expressions—such as *let your GOP folks to mask up*, *get him isolated and trace his close contacts* and *stay home since you felt it was not important enough to mask up*—shows the lack of consideration for others, as the implying intent is to treat GOP folks as stupid, less important and irresponsible. It is however important to highlight that there remains room to interpret the comments in excerpt 5 as somewhat uttered for the benefit of the addressee, especially if one sees these comments as an appropriate way to raise awareness on the issue of mask wearing, self-isolation and contact tracing, which all contribute to lifesaving in the context of a pandemic.

The antagonism around the appropriate conduct during the pandemic generated dismissive comments across the corpora. In excerpt 6, for instance, U_BBC_72 explicitly uttered “*get lost Rudi*,” whereas U_CNN_271 and U_CNN_377 as well as U_FOX_676 express their disinterest in the news about the diagnosis.

Excerpt 6

U_BBC_72: Get lost, Rudi …

U_CNN_271: Do you really think most **of us care**? Deal with it! / U_CNN_377: I could careless about Rudy what I do care about are the people he infected and that might die.

U_FOX_676: **Who cares**! Nobody advertises when I catch a virus!

The clash of expectations leads to an expression of disinterest in Giuliani’s diagnosis. One may, for example, argue that disregarding Giuliani’s illness this way is a marked behavior in the sense that understanding, showing compassion, and emotional support are the appropriate conduct in the context of a dreadful pandemic. However, it is important to highlight the variation in terms of the motives behind each dismissive comment. For example, unlike U_BBC_72 who uttered a short dismissing expression “*Get lost, Rudi*,” U_CNN_377 and U_FOX_676 elaborate on the reason for their dismissal of Giuliani. Concretely, while U_CNN_271 appears to be more concerned about people other than Giuliani, U_FOX_676 takes issue against the attention Giuliani’s test is getting.

Some reactions to Giuliani’s diagnosis focus on his involvement in attempting to overturn the results of the election. In excerpt 7, for instance, all the comments intend to silence Giuliani and have him kept away from the public eye. These users believe that the diagnosis is an opportunity not to hear Giuliani anymore.

Excerpt 7

U_BBC_409: So maybe, finally, at last Giuliani will shut up for a while at least

U_CNN_835: Someone should take him home and lock the doors.

U_FOX_242: Now he can quarantine 14 days and shut his lying mouth up…God works in mysterious ways…

Framing silencers such as shut up in a life-threatening context is certainly humiliating and aggressive, as it shows an explicit lack of consideration for Giuliani’s sense of worth and dignity. Undermining Giuliani’s sense of identity challenges the notion of social and moral normality which often motivates the expectation of sympathy in moments of despair. The absence of this sense of moral normality toward a suffering individual, thus, becomes indexical and marked, as this amounts to a failure to appropriately attend to others’ needs, which is an affront to the addresser and the addressee.

The polarization of views about the pandemic leads some users to question the seriousness of Giuliani’s diagnosis. In excerpt 8, U_CNN_364 and U_FOX_7 appear to encroach upon fellow users by downplaying the concern over Covid-19.

Excerpt 8

U_CNN_364: We all will get it sometime. It is what it is. Can’t shut the World down…

U_FOX_7: He will be fine. Lots of people I know have had it and been fine. 99% recovery rate sheeple. Stop letting the mainstream state run communist media lead you around by your short and curlies!

Whether these comments were expressed with panic-avoiding intentions through which U_CNN_364 and U_FOX_7 attempt to camouflage the seriousness and harmfulness of the pandemic, the use of such lexical stances as “*Can’t shut the World down*… and *Stop letting the mainstream state run communist media lead you around by your short and curlie*,” however, indexes the need to control fellow users’ way of thinking. As can be seen, the focus of the comments in excerpt 9 shifts from Giuliani’s diagnosis to the debate on how to behave in the midst of the pandemic.

Likewise, across Fox News and CNN a recurrent way to resurge the traditional political conflicts is through accusing users with opposing views as responsible for Giuliani’s positive diagnosis. In excerpt 9, for instance, U_CNN_119 and U_FOX_254 accuse the Democrats as responsible for Giuliani’s health shortcomings.

Excerpt 9

U_CNN_119: Democrats infected him because he’s been so successful in his efforts to over turn the election results! 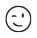

U_FOX_254: I’ve been saying for a while that leftist would find a way to get the virus to him.

Interestingly enough, other-blaming comments such as those in excerpts 9 are solely pervasive in Fox News and CNN corpora with zero incidence in the BBC corpus. One plausible explanation is that the main concern for the users commenting on the BBC page is the threat that the virus represents for Giuliani’s health and the rest of human beings, rather than the political divide of the US. Another explanation may be that, being a foreign news outlet, the users may not be as enthusiastic about engaging themselves in the promotion of conspiracy theories as are the US citizens themselves.

Some of the impolite comments are uttered with teasing intent so as to convey two opposing interactional meanings: prosocial (or non-aggressive) and antisocial (or aggressive) (Culpeper, 2011). In the former case, teasing is used for an affectionate, playful or joking end, whereas, in the latter case, teasing is used for a hostile, aggressive or malicious end. Excerpt 10 illustrates the interplay between both prosocial and antisocial ends. As U_BBC_284 utters “*Giuliani is filing a law suit in the Supreme Court to overturn his positive test 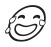
*” in which the user ends the comment by means of a face-with-tear-of-joy emoji, the teasing comment becomes a way of affectionately poking fun at Giuliani while enhancing positive feeling and relational quality among fellow users who may find the utterance amusing as well. Building upon this, the reactions of U285: *The best one yet*, U286: *lol* and U292: *kajajajaaaaaaaa* subscribes to the playful nature of this teasing. A similar example is found in CNN where U27 mockingly asserts “*he [Giuliani]’ll call the lab and tell them to overturn the results 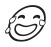

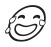

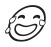
.*” As in the case of U_BBC_284, U_CNN_27 ends the comment with the suspension of three face-with-tear-of-joy emoji, generating laughter reactions from U_CNN_ 28 and U_CNN_30. Moreover, U_FOX_176 reuses Trump’s infamous phrase “*It will go away like a miracle*” with two face-with-tear-of-joy emoji 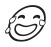

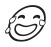
 so as to poke fun at Giuliani’s diagnosis. All these comments are classic provocative forms of social action that invite specific forms of response from participants (Haugh, 2017, p. 209). Against the prosocial end of these comments, however, not all the users found this humorous. U_BBC_889 is against poking fun as he/she aggressively alarms that there might be something wrong with all those who are happy when someone tests positive for Covid-19. It is likewise the belief of U_CNN_90 that making fun of others’ health condition is juvenile. Finally, U_FOX_209 conveys that it is abnormal and evil to laugh at others’ misfortune. For these users teasing comments become disrespectful forms of cruelty, which arguably amounts to social rejection and can result in emotional harm.

Excerpt 10

U284: Giuliani is filing a law suit in the Supreme Court to overturn his positive test 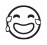/U_BBC_285 (Addressing U_BBC_284’s comment): The best one yet/ U_BBC_286 (Addressing U_BBC_284’s comment) lol. /U_BBC_295 (Addressing U_BBC_284’s comment) kajajajaaaaaaaa/U_BBC_889: What is wrong with you people that you are happy someone has covid, bunch of disgusting humans.

U_CNN_27: he’ll call the lab and tell them to overturn the results 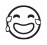

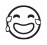

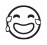
/ U_CNN_28 (Addressing U_CNN_27’s comment): I wouldn’t wish the virus on anyone, but your comment wins on the internet today! /U_CNN_29 (Addressing U27’s comment) I don’t think the situation is funny, but I did laugh……/U_CNN_30 (Addressing U_CNN_29’s comment): ***no you’re not. I laughed too. At this point, if we don’t laugh, we’ll cry and laughter is better for the heart and soul. Laugh on!/U90: Making fun of anybody’s health scare is juvenile. No matter who it is. . Be better people.***

U_FOX_176: Giuliani has been taken to the hospital. It will go away like a miracle. □ let us know how that works out for ya Rudy. /U_FOX_207: The flu will go away by Easter 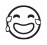
□
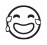
□/ U_FOX_209: Those laughing about this are obviously evil and vile!

The two ends of these teasing comments show how conflicts unfold across the corpora between sympathizers who perceive teasing comments as offensive, hurtful and evil, and detractors—willing to derive joy and pleasure at the expense of Giuliani’s health. It is also important to highlight the fact that the comments, as shown in excerpt 10, are all designed) in ways that invite some kind of affective response (or set of responses) on the part of fellow users (Haugh & Chang, 2019). These affective responses here range from amusement—displayed through the use of laughter—through to offense and anger. Calling those laughing as evil and vile certainly comes from anger, even if the comment is itself an impolite act for the use of offense generators (i.e., evil, vile).

At this point, there is no doubt that Giuliani’s diagnosis is a polarized topic about which the users are in a constant antagonism on how to react. This is why strategies such as agreement and disagreement are not just commonplace across the three corpora, but more importantly they play a substantial role in negotiating relationships. However, while agreement may build rapport between interactants, disagreement is generally accounted for as a face-threatening act which, depending on the degree, may cause conflict or simply damage the future dynamics of the interaction. In excerpt 11, for instance, U_BBC_303 discards the need to offer sympathy to Giuliani. However, the reactive comment made by U_BBC_307 to U_BBC_303’s act offers an opposing opinion. As a way of avoiding any conflictual misunderstanding, U_BBC_307 starts the comment in a way that seems to approve U_BBC_303’s utterance in an effort to mitigate the impact of the disagreement, before elaborating on his/her differing opinion by giving the reason for the disagreement with the hedge “*but maybe.*” U_BBC_307’s disagreeing comment shows to some extent the consideration U_BBC_307 has with respect to U_BBC_303’s feeling. Another example of disagreement is found in CNN where U_CNN_159 expresses disagreement over the efficacy of mask wearing in preventing the spread of the virus. Likewise, in Fox News, U_FOX_148 questions the relevance of mask wearing and hand washing in preventing from catching the virus, pointing to the fact he/she had already been diagnosed with the virus even after having followed these guidelines. U_FOX_149, however, offers a different perspective in the mask-wearing discussion, by firstly expressing the explicit disagreement (i.e., *That is incorrect*), before elaborating on his/her reasons which rests on the effectiveness of surgical masks in reducing the chance of transmission.

Excerpt 12

U_BBC_303: … Deserves no sympathy."/ U_BBC_ 307(Addressing U_BBC_303’s comment): *quite true, but maybe for humanity’s sake, we shouldn’t rejoice.*

U_CNN_159: *Rest giuliani. Standing up for the truth, rooting out the wrong*, ***his mask wouldn’t have stopped the spread of covid anyway***. *Show other places where it’s working. Display the standardization of mask use and design nationwide.*

U_FOX_148: *That’s his choice but it’s still not funny for an elderly person to get the virus. I wear a mask, wash my hands religiously and I still got it months ago. Masks really don’t do much, but we should at least try*

*U149: (Addressing U_FOX_148)* ***That is incorrect***. *Surgica masks reduce the chance of transmission of particles containing viruses from people between 70% and 98.5%. Their effectiveness is VERY high…*

One fact that stands out in the way users utter the comments is that the disagreement does not seem to restrict addressees’ action-environment as it would be the case in a power-asymmetric environment (see Locher, 2010). In other words, the sequences of disagreements found in the corpora do not necessarily call for some kind of reaction from the party disagreed with. I found no sequences across the corpora in which the disagreement between two users is framed in a back-and-forth manner that could amount to confrontation and conflict even though there is a clear clash of interests and goals in every instance where disagreement occurs. Perhaps this has to do with both the digital and multi-participant natures of this interaction.

### Impoliteness as the Struggle Over the Norms of Interaction (First Order)

As pointed out at the outset, impoliteness rests on individuals’ perceptions of (in)appropriate behavior. This perception is achieved through speech acts (i.e., complaint, criticism, etc.) considered to be social actions with implication on the (co-) constituting relation among individuals. In this subsection, the focus is on the first-order interpretation of the flow on the interaction across the corpora.

In excerpt 12, the intent of the three comments is not to react to Giuliani’s diagnosis per se; rather they are reactions to the behavior underlining the interventions of fellow users. In other words, the mere belief in these three comments is that there are comments within each corpus that are evaluated by U_BBC_89, U_CNN_153, and U_FOX_93 as sarcastically expressing joy and happiness about Giuliani’s diagnosis. These users’ understanding of the diagnosis is that Covid-19 is an issue to take seriously, and no one should joke about another person’s positive diagnosis as has been the intention of some comments within the thread. Additionally, there is another commonly shared element attached about these comments; that is, they are not just commenting on the disease as such, but they target the contents of what has been uttered in the preceding comments. In other words, the targets of these comments are fellow users within the interaction. Moreover, the fact that these three metapragmatic comments occur at turns 89 (BBC News), 153 (CNN), and 93 (Fox News) respectively suggests that these users had read and understood the preceding comments, and from this understanding, they take aim at fellow users’ interventions so as to protect Giuliani’s dignity as a human being.

Exceprt 13

U_BBC_89: What is wrong with you people that you are happy someone has covid.

U_CNN_153: These comments are hilarious. I have to admit they made me laugh. However Covid is not funny at all and I hope the dude gets well. Unfortunately, Im sure he’s spread this to MANY others and some people don’t have stellar healthcare access.

U_FOX_93: Really don’t know how people who are reacting to this with laughter can honestly live with themselves. It’s not just your own countryman who’s come down with #COVID19 but your fellow man too and you all find it amusing. How can you people be so callous…

The awareness in the minds of the lay users of what is the appropriate behavior under the circumstance of positive Covid-19 diagnosis is evident. Such appropriate conduct implies that Covid-19 is no laughing matter. The comments are an acknowledgment of the dramatic loss of human life worldwide and the reminder that the risk for anyone (including those engaged in the interaction) to get infected or potentially die from this virus is real. This metapragmatic accounts, on the other hand, for the epistemological fact that what is really at play in the three corpora is the struggle over the norms of interaction. Some users find Giuliani’s diagnosis as an issue akin to compassion, whilst others take the situation as a gratifying opportunity to generate laughter and happiness.

## Conclusions

This paper set out to examine impoliteness in Facebook generated by Giuliani’s Covid test and provide empirical responses to the following questions: How does impoliteness unfold in Facebook comments triggered by Giuliani’s diagnosis? On what grounds are the reactions to the diagnosis based across the three news outlets? An examination of the comments reveals that the reactions to Giuliani’s diagnosis are more focused on him being a politician, rather than him being a human being affected by the virus. The reactions attack his actions and the actions of others within his political party, which suggests that impoliteness in this context has a strong dependence on previous actions and political engagement. Giuliani is seen to be undeserving of compassion or empathy not just on the grounds of his active involvement in attempting to overturn the presidential election results (see excerpt 10), but also due to his disregard toward mask wearing in public spheres. Political polarization is, in other words, what shapes and motivates the negative reactions in this context. Not all the users, however, appreciate the attacks targeting Giuliani. Through metadiscursive comments, some users not just feel the need to treat Giuliani as a human being, but more importantly remind fellow users that Covid-19 should be a concern for all. What is particularly critical about these metacomments is that while the users advocate for civil interactions, they mostly do not condone Giuliani’s actions. The reason for this is arguably the salience of the moral order which implicitly shapes these users’ understanding of what should be obligatory, permissible, or forbidden under the circumstances, regardless of Giuliani’s prior actions. These users ground their awareness of what should be the appropriate conduct in a situation of despair such as dealing with Covid-19 and describe fellow users’ conducts as the type of behavior against which they identify themselves. How they see themselves as human beings is different from how they see others. Comments conveying a laughing attitude are, for instance, metapragmatically singled out as being discourteous and impolite (see excerpt 13).

The findings also further our understanding of the increasing polarization of social political discourse on Facebook (Bou-Franch, 2021; Tagg, 2017) and its direct relations to the Covid-19 pandemic (Andersson, 2022). The reactions to Giuliani’s diagnosis show how the opposition between “us” and “them” turns an issue of life or death into an opportunity to resurge traditional political divisions, making the diagnosis becomes a real opportunity for “us” to compete against “them.” In excerpt 1, for instance, the insulting comment posted by U_CNN_201 takes aim at the Republican leadership for their supposed mismanagement of the pandemic. In excerpt 10, U_FOX_176 reuses Trump’s infamous phrase “it will go away like a miracle” as a way to poke fun at Giuliani’s expense. These comments show how individuals can overlook delicate situations and be bluntly dismissive of other individuals in need for political interests. These behaviors do not however go unsanctioned on the grounds of what is morally right and wrong. The pervasiveness of metacomments in these translocality environments calling out marked behaviors and interpreting the context of the interaction and the situation itself as something which requires respect, understanding and compassion shows that even in open-ended platforms there is an implicit expectation grounded on the “seen but unnoticed” (Haugh, 2013); that is, the idea that behaving politely or understanding someone in a delicate situation is what every member of a society is entitled to know or describe and communicate. As a social practice, impoliteness rests not just on individuals’ perception of themselves as social beings, their expectations grounded on the difference between right and wrong as well as the position they take in a given interaction, but it also depends on an implicit shared understanding of the values and meaning of the interaction as a representation of a given society.

Furthermore, when looking into the nature per se of impolite comments across the three corpora, the revelation is that there is a difference in terms of the orientations of the attacks and how the users portray Giuliani. For instance, most criticisms in the CNN and the BBC corpora merely convey two main sets of concerns. On the one hand, users question the seriousness and integrity of Trump in delivering the news about Giuliani’s diagnosis, presupposing Trump to be a liar and untrustworthy. The second set of comments in CNN and BBC corpora are not merely concerned about Giuliani’s health; rather they tend to raise concerns about the safety of the people with whom Giuliani may have been in contact, raising the question of whether those people are safe. However, within Fox News, most comments tend to express doubts over the fact that Republican Politicians are the only politicians being infected by the virus, raising suspicion and conspiracies. This difference in orientation of the intentions is a revelation of not just the political polarization of users’ views but, more importantly, it opens the window to how the coronavirus pandemic has shaped the political expressions of Facebook users.

The roles of participants in these interactions are also salient. Not only there are varied targets the reactions orient to, but mostly, the users perceiving or evaluating comments as offensive, which is conveyed through metadiscursive comments, are individuals other than Giuliani himself. The impolite meaning arises here not because the main targets are either offended or not, but because the interpretation of comments as offensive rests on the third party’s understanding of such offense through talk-in interaction. This stresses the view that impoliteness evaluation is not a matter of the dyadic addressee-addresser alone (Eelen, 2001), rather it is the bystanders’ interpretation and evaluation, supporting Haugh’s (2013, p. 56) argument that the “evaluation of impoliteness needs to be situated vis-à-vis not only simply speakers or hearers, but also relative to a complex array of production and reception footings, the co-constitution of which is itself and morally implicative activity in interact.”

Moreover, the findings as presented in this paper go along with what has already put forward in previous studies on individuals’ reactions to Covid-19 on Facebook. This is not just in terms of stressing the importance of political ideology in shaping individual’s issues such as mask wearing, but also in highlighting the tendency to voicing emotion that reinforces their dissatisfaction with the way the opposing group (re)acts vis-à-vis the Covid-19 emergency (Andersson, 2022). This reiterates both the situatedness of the norms of interaction and the (co-)construction of the identities of the interactants through impoliteness behaviors in polarized contexts. In terms of the situatedness of the norms of the interaction, impoliteness behaviors analyzed here are social actions that are situated within a particular social or relational network (Bou-Franch, 2021; Haugh & Kádár, 2013; Tsoumou, 2022). The meaning of impoliteness in these corpora is connected to breaches in appropriate behavior regarding the topic management and social expectations associated with the impact of Covid-19. In terms of identity (co-)construction, impoliteness here plays a role in the way interactants tend to position themselves with respect to others (Andersson, 2021). Just as Andersson (2022) reported, the findings in this paper show that offensive language marks and negotiates different value positions in Facebook interaction about Covid-19.

Finally, the present paper is certainly limited in terms of its focus as it examines impoliteness alone, without considering politeness comments. With the understanding that impoliteness in a context of life-or-death situation is just part of the overall practice, future research shall explore the polite comments so as to see both their contents as well as their forms, and contrast the results. It would also be interesting to explore similarities and differences of the present findings in other platforms. Finally, the relevance of classic sociolinguistic variables of the users as well as their competence in English should be explored in future research so as to determine the connection between these variables and impoliteness practices on Facebook.
